# Menopause symptom awareness research across the globe: a scoping review

**DOI:** 10.3389/fgwh.2026.1829378

**Published:** 2026-05-28

**Authors:** E. Williams, L. Terry, M. Lindeman, M. E. Wright

**Affiliations:** 1 School of Medicine, Cardiff University, Cardiff, United Kingdom; 2 School of Optometry and Vision Sciences, Cardiff University, Cardiff, United Kingdom; 3 School of Geographical Sciences, University of Bristol, Bristol, United Kingdom; 4 Cardiff University Brain Imaging Research Centre (CUBRIC), Cardiff University, Cardiff, United Kingdom

**Keywords:** international, menopause, scoping review, symptom awareness, underrepresented

## Abstract

**Introduction:**

Menopause is a major global health burden that directly impacts the wellbeing of half the population. Menopause is associated with a range of psychological, vasomotor, somatic and urogynocological symptoms that impact quality-of-life and day-to-day functioning. Despite this, it is still an under-discussed and taboo topic internationally, with women reporting a lack of understanding and social support, which in turn worsens the symptom experience. A lack of awareness or knowledge of menopause symptoms is a barrier to effective and understanding social support. Therefore, it is important that not only those going through menopause have awareness of symptoms, but those within their social and professional networks. To ease the global impact of menopausal symptoms, a vital step is raising awareness and encouraging social support. This scoping review aims to map current research into menopause symptom awareness across the world, sampling all ages and genders in the non-medical general public in low- to high-income countries.

**Methods:**

Online databases were used to identify eligible studies up to June 2025, of which 84 were selected for the final sample.

**Results and discussion:**

Prevalence of research across the world is mapped and underrepresented areas are highlighted, including non-female samples, low-income countries, and assessment of ocular symptom knowledge. This work will inform and highlight priority areas for future research and awareness campaigns.

**Systematic Review Registration:**

https://doi.org/10.17605/OSF.IO/CT5V6.

## Introduction

Menopause is an endocrine transition and reproductive milestone that will directly impact around half of the global population. By 2030 there will be an estimated 1.2 billion menopausal women around the world (1). It is associated with a range of psychological, vasomotor, somatic, urogynocological and ocular symptoms [ (2, 3); for examples of the range of potential menopause symptoms, see Table 1], which can dramatically impact quality-of-life and day-to-day functioning (4–6). The impact of these symptoms can be amplified by social stressors that typically coincide with the menopause age range, such as shifting caregiving responsibilities and workplace pressure, causing significant distress (7–9).

**Table 1:** A non-exhaustive breakdown of potential menopausal symptoms.

Domain	Example symptoms
Psychological	Difficulty concentrating, memory loss, irritability, depressed mood
Vasomotor	Hot flushes, night sweats, sleep disturbances
Somatic	Joint/muscle pain, heart palpitations, fatigue, headaches
Urogynocological	Vaginal dryness/irritation, itching, recurrent urinary tract infections, dyspareunia, dysuria
Ocular	Dry eye, changes to light sensitivity

Social support and high education about menopause have been found to be associated with women perceiving their symptoms as less severe (10, 11) and improves perceived ability to deal with these symptoms (12, 13). However, a prevalent lack of awareness or knowledge of menopause symptoms has been reported across cultures (14) and sexes [e.g. (15–17),] and may be a barrier to effective and understanding social support. This may be amplified in certain symptom domains; for example, ocular symptoms, such as vision changes, light sensitivity and dry eye, are common when specifically investigated (18, 19) yet rarely mentioned when surveying about menopause knowledge. It is essential that not only those going through menopause have an awareness of symptoms and their impact, but also those within their social and professional network so adequate support can be provided.

An important consideration is that prevalence of menopause symptomatology, education and social perception differ greatly between cultures and countries (20), which will impact women's experiences and population awareness. To guide global awareness campaigns, it is therefore vital that menopause symptom awareness and contributing factors are appraised globally, so menopause education initiatives can be tailored to cultures and areas of need, improving healthcare inequities.

This scoping review aims to investigate the landscape of existing research into menopause symptom awareness globally, across sex and age, including which countries and populations are not yet well represented in the literature. This will highlight prevalent gaps in scientific knowledge and help to guide future research and awareness campaigns.

## Methods

### Primary review question

The primary aim of this scoping review was to investigate how much original research has been conducted into menopause symptom awareness globally. Specifically:

- What is the proportion of literature investigating menopause symptom awareness in different countries/global regions?
- What is the proportion of this literature that has been cross-cultural?

### Secondary review question

In research that has explored menopause symptom awareness:

- Which populations have been studied?
  ○ What is the representation of low-, middle- and high-income countries?
  ○ What is the representation of demographics (e.g., socioeconomic status, education) amongst the participants?
  ○ What is the proportion of studies that included cisgender men or gender diverse populations (either incidentally or as the main focus of research)?
- Which symptoms or symptom areas were included?
- What was the research or study design? (i.e., is the outcome quantitative or qualitative?)
- How has the quantity and design of research changed over time?
- What levels of symptom awareness are reported by region and gender?

### Registration

This manuscript has been written based on the extension of the Preferred Reporting Items for Systematic Reviews and Meta-Analysis for Scoping Reviews (PRISMA-ScR) (21). This protocol was written before the review activities and registered on Open Science Framework (OSF) on 04/07/2025.

### Search strategy

Searches were conducted on the electronic databases Ovid MEDLINE, PubMed and Web of Science with the search terms outlined in Table 2, including all publications from inception to June 2025. The exclusion and inclusion criteria are outlined in Table 3. Boolean operators were used to search for studies with menopausal and awareness key terms in titles and abstracts, and to exclude ineligible study design types.

**Table 2:** Table of keywords used for the three stages of literature review. Search terms were combined using Boolean operators (AND, OR) to include various combinations of keywords. Wildcards (*) were used to capture variations of terms, such as “menopaus*”.

Menopause terms	Boolean operator	Awareness terms	Boolean operator	Study type
(Menopaus*) or (climacteric) or (perimenopaus*) or (postmenopaus*)	and	(Knowledge) or (awareness) or (understand*) or (perception)	not	(Review) or (comment) or (editorial) or (letter) or (lecture) or (systematic review) or (scoping review) or (book)

**Table 3:** Full inclusion and exclusion criteria.

Criterion	Include	Exclude
Population	Studies of awareness of menopause in all people including menopause-relevant cohorts (surgical, medical, and natural menopause, premenopausal, perimenopausal, and postmenopausal), cisgender men, gender diverse individuals and younger cohorts (children and adolescents).	Studies that focus solely on healthcare practitioners’ awareness or knowledge.
Comorbidity	Different types of menopause, including premature and surgical menopause.	Comorbid relationships such as the interaction between menopausal symptoms and HIV or cystic fibrosis.
Awareness	Studies of awareness or knowledge about peri and post-menopause symptoms and experience.	Studies focused on hormone replacement therapy symptom alleviation awareness.
Study design	Primary research papers (qualitative or quantitative), describing original research into menopause/menopause symptom awareness.	Systematic reviews, comments, editorials, letters, lectures, scoping reviews, literature/narrative reviews, case studies, books. *In-vitro* studies, animal studies. Descriptions of protocols or similar that do not describe original relevant data.
Language	Papers written in English or with a translated version provided by the publisher.	Papers without a translation into English.

### Study screening and data extraction

Duplication deletion, title/abstract screening, and full text screening was completed using the Rayyan online software (Rayyan Systems Inc., Cambridge, MA, USA) (22). The titles and abstracts were independently screened for eligibility by at least one reviewer (EW or MEW); ∼10% of study abstracts were screened by both reviewers to ensure agreement. The full text was then screened for inclusion by at least two reviewers (EW, ML, and/or MEW). For included studies, the data were extracted using a standardised data extraction sheet by one reviewer (either EW or ML; for the contents of the data extraction sheet, see Supplementary Material). Data extraction from 10% of these final studies were completed by two reviews to compare outputs and ensure agreement. Any differences at any point in this process were resolved with a discussion between the whole reviewer team (EW, LT, ML, and MEW).

### Analysis and figure generation

Results are described narratively, and appropriate descriptive statistics and graphics used to address our research questions. If a study sampled more than one country/continent, a datapoint was added for each region for relevant sections of the results. These were further split into demographical information (e.g., year of publication, socioeconomic status, methodology) relevant to our secondary research questions. Average awareness reported in each study was also extracted and given a numerical code (0 = Unclear,1 = low, 2 = mostly low, 3 = moderate/mostly moderate/mixed, 4 = mostly high, 5 = high). All figured are plotted in Rstudio (23, 24).

## Results

### Search results

**Figure 1:**
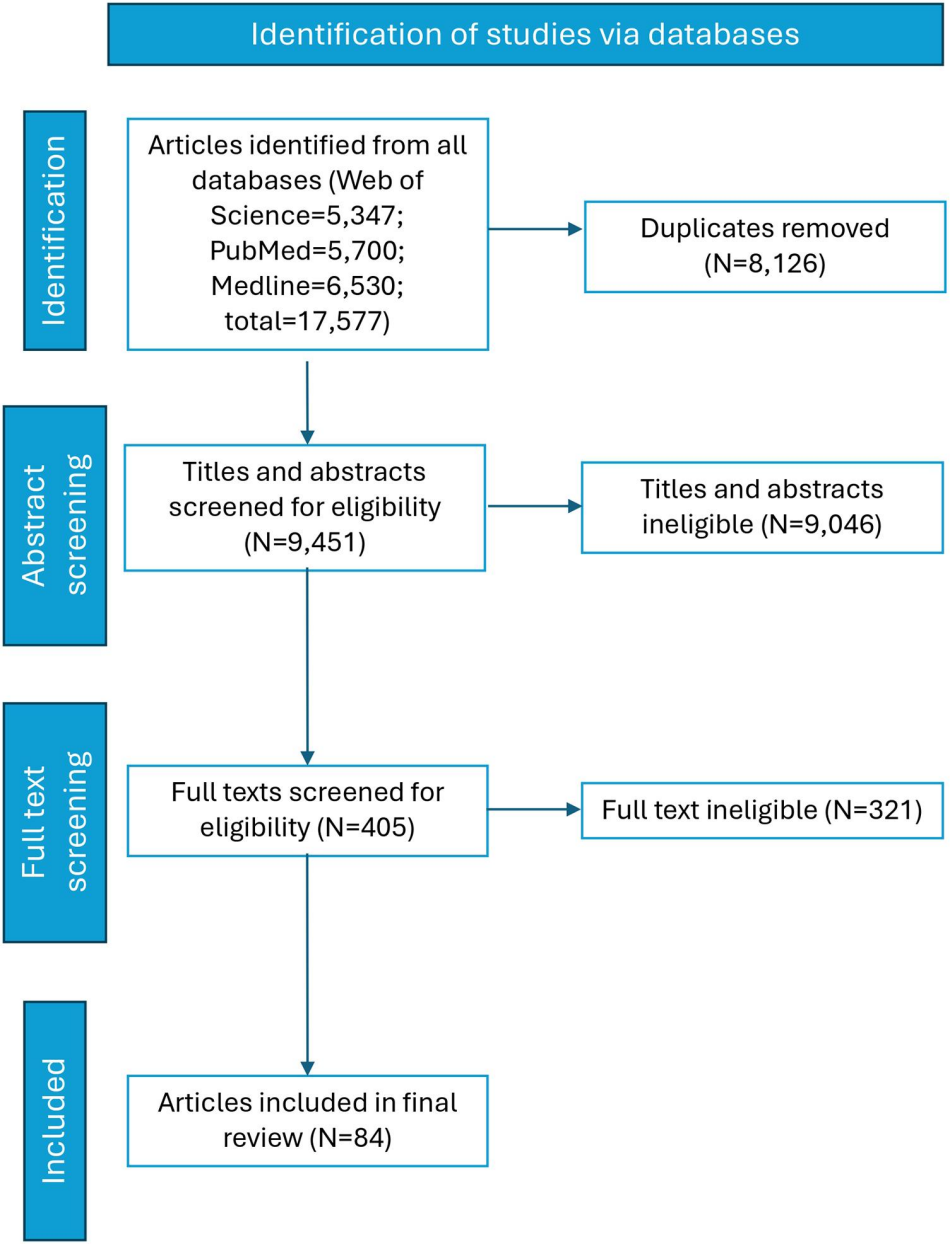
Preferred reporting Items for Systematic Reviews and Meta-Analyses (PRISMA) flow diagram presenting the process of study identification, screening, and selection of studies.

The database search identified 17,577 studies using the above keywords (Table 2), of which 8,126 were duplicates and therefore removed. After abstract and full text screening, 9,367 articles were removed due to not meeting the eligibility criteria. A total of 84 studies were included in the final stage of the review (see Figure 1).

### Study characteristics

84 studies met the criteria to be included in the final review, published between 1980 and 2025 (see Table 4). This included 65 with quantitative approaches, 14 with qualitative, and 5 with mixed methodologies. Not all studies had a clear definition of menopause (if menopausal women were included), but the most common definition was amenorrhea for 6–12 months. A smaller number emphasised hormonal decline or the lack of fertility. Sample sizes ranged from 15 to 2509.

**Table 4:** Details of included studies - study description of all included studies. Age groups refer to the majority of the sample to aid in cross-study comparison. International Organisation for Standardisation (ISO) country and continent codes used.

Study	Country code	Continent code	No. Of countries sampled	Age	Gender identities included	Socioeconomic/education status	Menopause status	Specific symptom domains assessed?	Quantitative/ Qualitative	Awareness level
(56)	SA	AS	1	Mid reproductive life (25–40)	All women	Mostly moderate	Menopausal included	Yes	Mixed	Moderate
(57)	IR	AS	1	Late reproductive life (40–60)	All women	Mostly low	All peri/post menopausal	Unclear	Quantitative	Mostly moderate
(58)	PK	AS	1	Late reproductive life (40–60)	All women	Not reported	Menopausal included	Unclear	Quantitative	Mix
(59)	SA	AS	1	Late reproductive life (40–60)	All women	Mostly high	Menopausal included	Yes	Quantitative	Low
(60)	JO	AS	1	Mid reproductive life (25–40)	All women	Mostly moderate	None menopausal	Yes	Quantitative	Moderate
(61)	NG	AF	1	Late reproductive life (40–60)	All women	Equal mix	All peri/post menopausal	Yes	Quantitative	Mix
(62)	IR	AS	1	Late reproductive life (40–60)	All men	Mostly low	None menopausal	Unclear	Quantitative	Low
(63)	PK	AS	1	Lifespan	All men	Equal mix	None menopausal	No	Quantitative	Low
**(** 49 **)**	IN	AS	1	Late reproductive life (40–60)	All women	Mostly moderate	All peri/post menopausal	Yes	Quantitative	Mix
(64)	ES	EU	1	Lifespan	All women	Equal mix	Menopausal included	Yes	Qualitative	Mix
(65)	SA	AS	1	Late reproductive life (40–60)	All women	Mostly low	Status not defined	No	Quantitative	Low
(66)	NG	AF	1	Late reproductive life (40–60)	All women	Mostly moderate	None menopausal	Yes	Quantitative	Mostly low
(67)	SE	EU	1	Late reproductive life (40–60)	All women	All moderate	All peri/post menopausal	No	Qualitative	Moderate
(68)	IN	AS	1	Late reproductive life (40–60)	All women	Not reported	None menopausal	No	Quantitative	Moderate
(69)	CA	NA	1	Late reproductive life (40–60)	All women	Mostly high	All peri/post menopausal	Yes	Quantitative	Mostly moderate
(70)	ES	EU	1	Late reproductive life (40–60)	All women	Mostly moderate	All peri/post menopausal	No	Qualitative	Low
(71)	ES	EU	1	Late reproductive life (40–60)	All women	Not reported	All peri/post menopausal	No	Quantitative	Moderate
(72)	BR	SA	1	Late reproductive life (40–60)	All women	Not reported	All peri/post menopausal	Unclear	Qualitative	Moderate
(73)	BR	SA	1	Late reproductive life (40–60)	All women	Equal mix	Menopausal included	No	Qualitative	Mostly moderate
(73)	BR	SA	1	Late reproductive life (40–60)	All women	Equal mix	Menopausal included	Yes	Quantitative	Mix
(74)	SA	AS	1	Late reproductive life (40–60)	All women	Mostly high	Menopausal included	Yes	Quantitative	Low
(75)	TR	AS	1	Mid reproductive life (25–40)	All women	All moderate	Menopausal included	Yes	Quantitative	Low
(76)	IT	EU	1	Late reproductive life (40–60)	All women	Equal mix	Menopausal included	Yes	Quantitative	Mix
(50)	IN	AS	1	Late reproductive life (40–60)	All women	Equal mix	All peri/post menopausal	Yes	Quantitative	Low
(77)	IR	AS	1	Late reproductive life (40–60)	All women	Mostly low	Menopausal included	Yes	Quantitative	Mostly high
(78)	ES	EU	1	Late reproductive life (40–60)	All men	Mostly high	None menopausal	Yes	Quantitative	Mostly moderate
(79)	IR	AS	1	Late reproductive life (40–60)	All women	Mostly high	All peri/post menopausal	No	Quantitative	Mix
(80)	BF	AF	1	Late reproductive life (40–60)	All women	Mostly low	All peri/post menopausal	No	Qualitative	Low
(81)	AU	OC	1	Late reproductive life (40–60)	All women	Mostly low	Menopausal included	Yes	Quantitative	Low
(39)	ER	AF	1	Late reproductive life (40–60)	All women	Mostly moderate	Status not defined	Yes	Quantitative	Mostly high
(82)	US	NA	1	Late reproductive life (40–60)	All women	Mostly moderate	All peri/post menopausal	No	Qualitative	Unclear
(83)	NL	EU	1	Late reproductive life (40–60)	All women	Mostly high	Menopausal included	Yes	Quantitative	Moderate
(53)	HK, CN	AS	2	Lifespan	All women	Mostly low	Menopausal included	Unclear	Quantitative	Low
(84)	AE	AS	1	Late reproductive life (40–60)	All women	Equal mix	Menopausal included	Yes	Quantitative	Mostly moderate
(85)	GB	EU	1	Late reproductive life (40–60)	Women and men	Mostly moderate	Status not defined	Unclear	Quantitative	Moderate
(86)	BD	AS	1	Late reproductive life (40–60)	All women	Mostly moderate	All peri/post menopausal	Yes	Quantitative	Mix
(87)	GB	EU	1	Young adulthood (18–25)	Women and men	Mostly moderate	Status not defined	No	Qualitative	Mostly low
(88)	AU	OC	1	Late reproductive life (40–60)	All women	Mostly moderate	Menopausal included	No	Qualitative	Mix
(89)	TR	AS	1	Late reproductive life (40–60)	All men	Mostly low	None menopausal	No	Qualitative	Low
(90)	US	NA	1	Late reproductive life (40–60)	All women	Mostly low	All peri/post menopausal	No	Quantitative	Mostly low
(91)	BH	AS	1	Late reproductive life (40–60)	All women	Equal mix	Menopausal included	Yes	Quantitative	Low
(92)	US	NA	1	Young adulthood (18–25)	All women	All high	None menopausal	Yes	Quantitative	Low
(93)	TR	AS	1	Late reproductive life (40–60)	All women	Mostly low	Menopausal included	Unclear	Quantitative	Mostly moderate
(94)	PL	EU	1	Lifespan	All women	Mostly moderate	None menopausal	Yes	Quantitative	Mostly low
**(** 95 **)**	US	NA	1	Late reproductive life (40–60)	All women	Mostly low	Menopausal included	Yes	Quantitative	Low
(96)	GB	EU	1	Late reproductive life (40–60)	All women	Equal mix	None menopausal	Unclear	Quantitative	Mostly low
(97)	SE	EU	1	Late reproductive life (40–60)	All women	Equal mix	All peri/post menopausal	Yes	Quantitative	Mostly low
(98)	SG	AS	1	Late reproductive life (40–60)	All women	Not reported	Menopausal included	No	Qualitative	Low
(99)	PK	AS	1	Late reproductive life (40–60)	All women	Mostly low	All peri/post menopausal	Unclear	Quantitative	Low
(100)	SA	AF	1	Late reproductive life (40–60)	All women	Equal mix	Menopausal included	Yes	Quantitative	Low
(101)	US	NA	1	Late reproductive life (40–60)	All women	Equal mix	All peri/post menopausal	Unclear	Quantitative	Mostly low
(102)	JP	AS	1	Late reproductive life (40–60)	All women	All high	Menopausal included	No	Quantitative	Mostly high
(103)	PK	AS	1	Late reproductive life (40–60)	All women	Mostly low	All peri/post menopausal	No	Quantitative	Low
(104)	CD	AF	1	Late reproductive life (40–60)	All women	Mostly moderate	Menopausal included	Yes	Quantitative	Low
(105)	IN	AS	1	Late reproductive life (40–60)	All women	Mostly moderate	All peri/post menopausal	No	Qualitative	Low
(106)	IQ	AS	1	Late reproductive life (40–60)	All women	Mostly low	All peri/post menopausal	Unclear	Quantitative	Mostly moderate
(54)	AR, BR, CL, CO, MX	SA, NA	5	Older adulthood (60+)	All women	Mostly high	All peri/post menopausal	Yes	Quantitative	Mix
(107)	IR	AS	1	Late reproductive life (40–60)	All women	Mostly moderate	None menopausal	Yes	Quantitative	Moderate
(108)	IR	AS	1	Late reproductive life (40–60)	All women	Mostly low	All peri/post menopausal	Unclear	Quantitative	Mostly moderate
(109)	PK	AS	1	Late reproductive life (40–60)	All women	Mostly low	Menopausal included	No	Quantitative	Low
(110)	BR	SA	1	Late reproductive life (40–60)	All women	Equal mix	Menopausal included	Yes	Quantitative	Mix
(111)	TW	AS	1	Lifespan	All women	Mostly moderate	Menopausal included	Yes	Mixed	Low
(112)	US	NA	1	Late reproductive life (40–60)	All men	Equal mix	None menopausal	Yes	Quantitative	Mostly low
(15)	CA	NA	1	Young adulthood (18–25)	Non-binary, women, men	All high	Status not defined	Yes	Quantitative	Moderate
(113)	NP	AS	1	Late reproductive life (40–60)	All women	Equal mix	Menopausal included	Yes	Quantitative	Mostly low
(114)	LK	AS	1	Late reproductive life (40–60)	All women	Not reported	All peri/post menopausal	Unclear	Quantitative	Moderate
(115)	BR	SA	1	Late reproductive life (40–60)	All women	Equal mix	All peri/post menopausal	Yes	Mixed	Mostly high
(116)	US	NA	1	Late reproductive life (40–60)	All women	Mostly low	All peri/post menopausal	Yes	Quantitative	Low
(117)	EC	SA	1	Late reproductive life (40–60)	All women	Mostly low	Menopausal included	Yes	Quantitative	Mostly low
(118)	AE	AS	1	Late reproductive life (40–60)	All women	Equal mix	Menopausal included	Yes	Quantitative	Mostly moderate
(16)	GB	EU	1	Lifespan	Women and men	Equal mix	Menopausal included	Yes	Quantitative	Moderate
(119)	IN	AS	1	Late reproductive life (40–60)	All women	Equal mix	All peri/post menopausal	Yes	Quantitative	Moderate
(120)	AE	AS	1	Late reproductive life (40–60)	All women	Mostly moderate	Menopausal included	Yes	Qualitative	Mostly moderate
(121)	JP	AS	1	Mid reproductive life (25–40)	All women	Equal mix	All peri/post menopausal	Yes	Quantitative	Moderate
(122)	CA	NA	1	Late reproductive life (40–60)	All women	Not reported	Menopausal included	No	Qualitative	Low
(123)	IR	AS	1	Late reproductive life (40–60)	All women	Equal mix	All peri/post menopausal	No	Quantitative	Moderate
(51)	TW	AS	1	Late reproductive life (40–60)	All women	Mostly low	Menopausal included	Yes	Quantitative	Low
(124)	TR	AS	1	Late reproductive life (40–60)	All women	Equal mix	All peri/post menopausal	No	Quantitative	Mostly moderate
(125)	IT	EU	1	Late reproductive life (40–60)	All women	Mostly low	Menopausal included	Yes	Mixed	Mix
(17)	IN	AS	1	Late reproductive life (40–60)	All men	Mostly low	None menopausal	Yes	Mixed	Low
(55)	US, CA	NA	2	Lifespan	Women and men	Not reported	Status not defined	No	Quantitative	Mix
(126)	ET	AF	1	Lifespan	All women	Mostly low	Menopausal included	Yes	Quantitative	Low
(127)	IR	AS	1	Late reproductive life (40–60)	Women and men	Mostly moderate	All peri/post menopausal	No	Quantitative	Mix
(128)	CN	AS	1	Mid reproductive life (25–40)	All women	Equal mix	None menopausal	Yes	Quantitative	Low

### Primary research question

#### How much research has been done into menopause symptom awareness globally?

**Figure 2:**
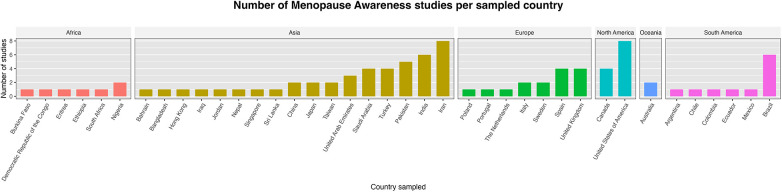
The counts of menopause symptom awareness studies for each country from the final sample (*N* = 84). If a study sampled more than one country/continent, a datapoint was added for each country.

**Figure 3:**
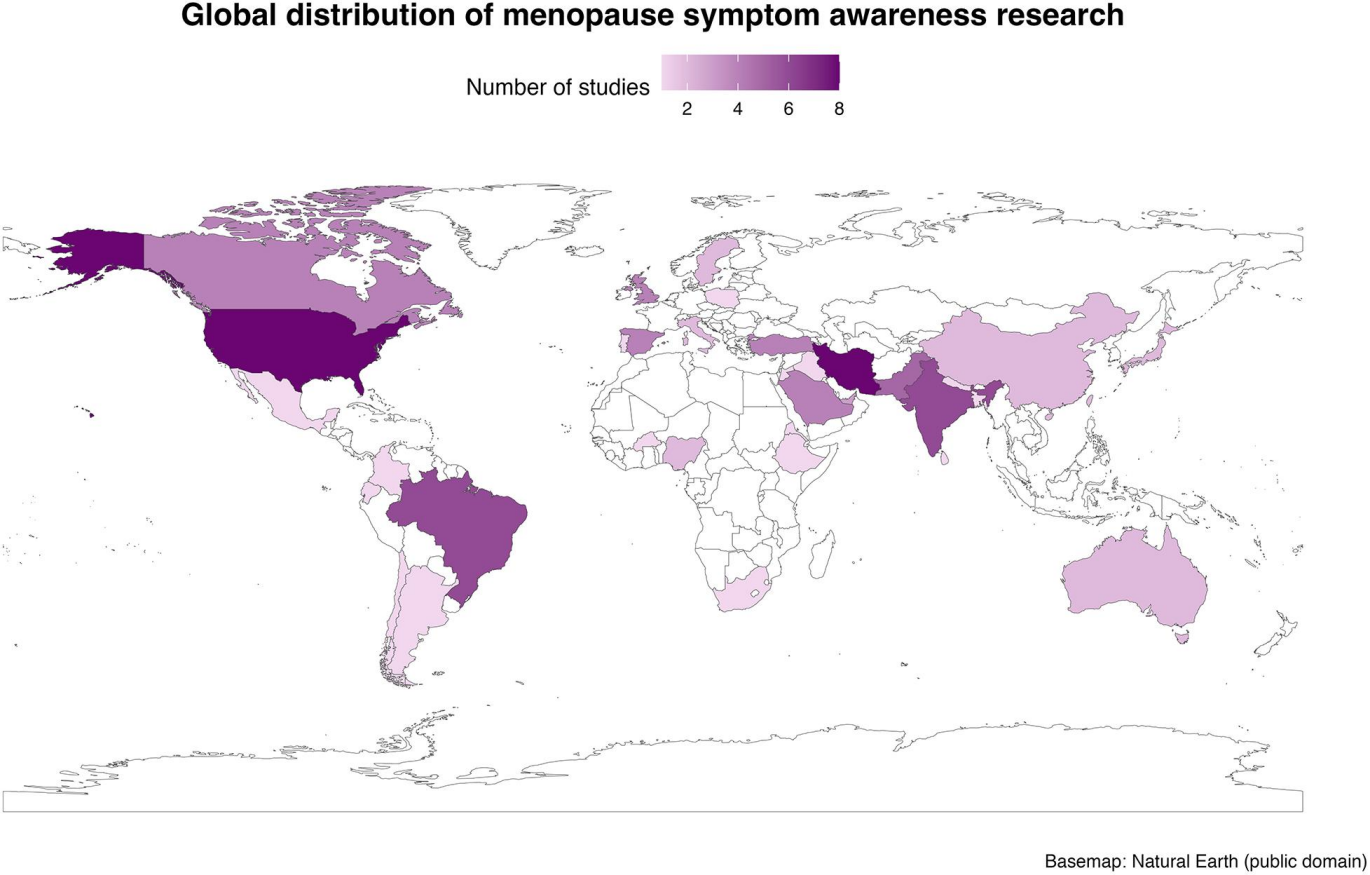
Illustration of the number of menopause symptom awareness studies per country in the final sample (*N* = 84) across a worldwide map.

The sampled countries, split into continents, are displayed in Figure 2 and mapped in Figure 3. The majority of studies were localised within a single country, with direct cross-cultural comparisons only possible in a minority; three studies sampled 2 countries and one sampled 5. Most research has so far been completed in Asian countries, notably Iran, India, and Pakistan, with a significant number of studies also being conducted in North America. Similar to other scientific areas [e.g. (25–27),], African countries were severely under-represented in this research, with only 7 studies (8.3% of the full sample) and 6 countries sampled. The least researched continent was Oceania; 2 studies sampled populations from Australia, while none focused on other regions, such as the Pacific islands.

### Secondary research questions

#### Which populations have been studied?

Sample focus was overwhelmingly on women-only populations (*N* = 72 studies). Men were included in only 12 studies, of which 6 were all-male and 6 were mixed gender. Of this latter group, just one study included non-binary participants (15). Out of those that sampled women, menopause status also varied but was mostly aimed at menopausal populations; 29 studies sampled exclusively menopausal participants, 35 mixed menopausal and non-menopausal, 8 sampled non-menopausal groups, and 6 did not define.

**Figure 4:**
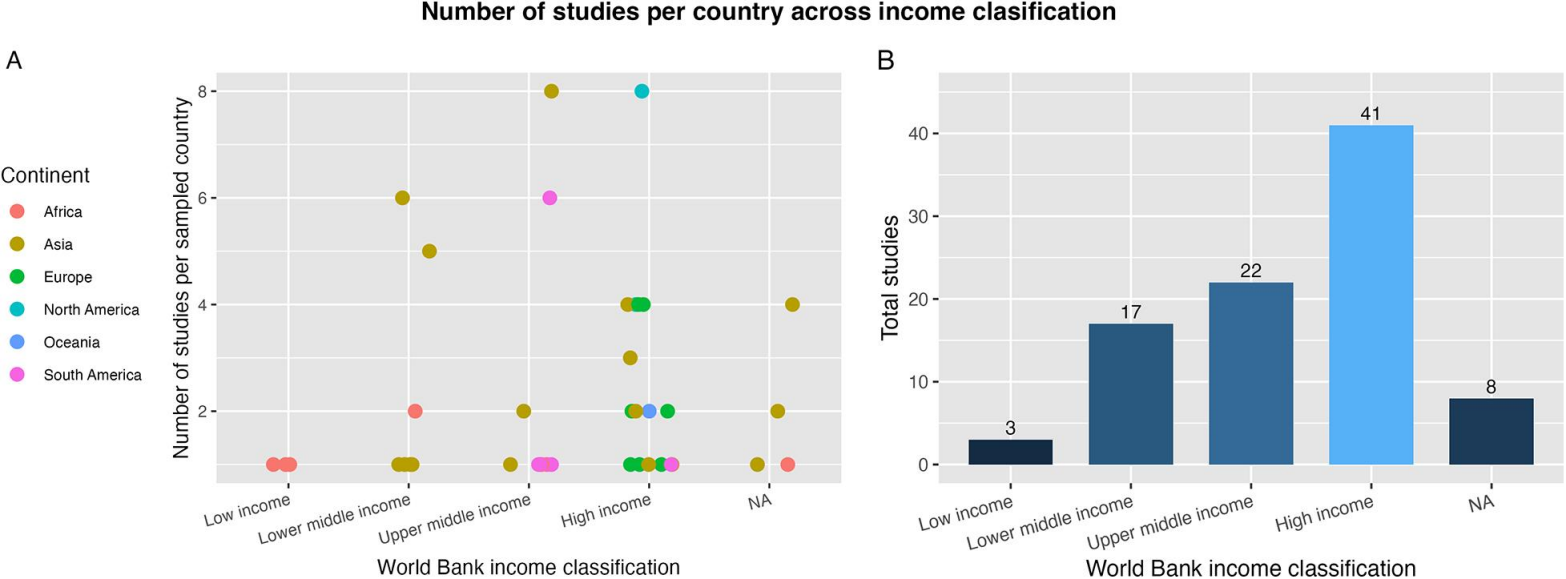
Illustration of the number of studies that sample differing income level countries, as classified by the World Bank (accessed February 2026). Those countries with no classification are labelled ‘NA’. Data is plotted as the number of studies in each country (colour coded by continent; panel **A**), or the total number of studies sampling countries in that income classification (panel **B**). Jitter in the *x*-axis direction has been added to panel A data points to aid visibility.

The spread of sampled countries was then examined by World Bank income classification (Figure 4; Data retrieved February 2026). Mirroring our primary findings, menopause symptom awareness research was heavily skewed toward middle- and high-income countries, with very few studies sampling populations from low-income regions.

#### Which symptoms or symptom areas were included?

47 papers explicitly mention specific symptom domains, which is ∼56% of the selected papers. Of these, physical symptoms were the most frequently mentioned (e.g., cessation of menstruation, weight gain; *N* = 43), followed by vasomotor symptoms (e.g., hot flushes; *N* = 39) and psychosocial symptoms (e.g., cognitive changes; *N* = 39), then sexual symptoms (e.g., loss of sexual libido; *N* = 31). Finally, ocular symptoms, such as dry eye or light sensitivity changes were the least represented (*N* = 4).

#### How has the quantity and design of research changed over time?

**Figure 5:**
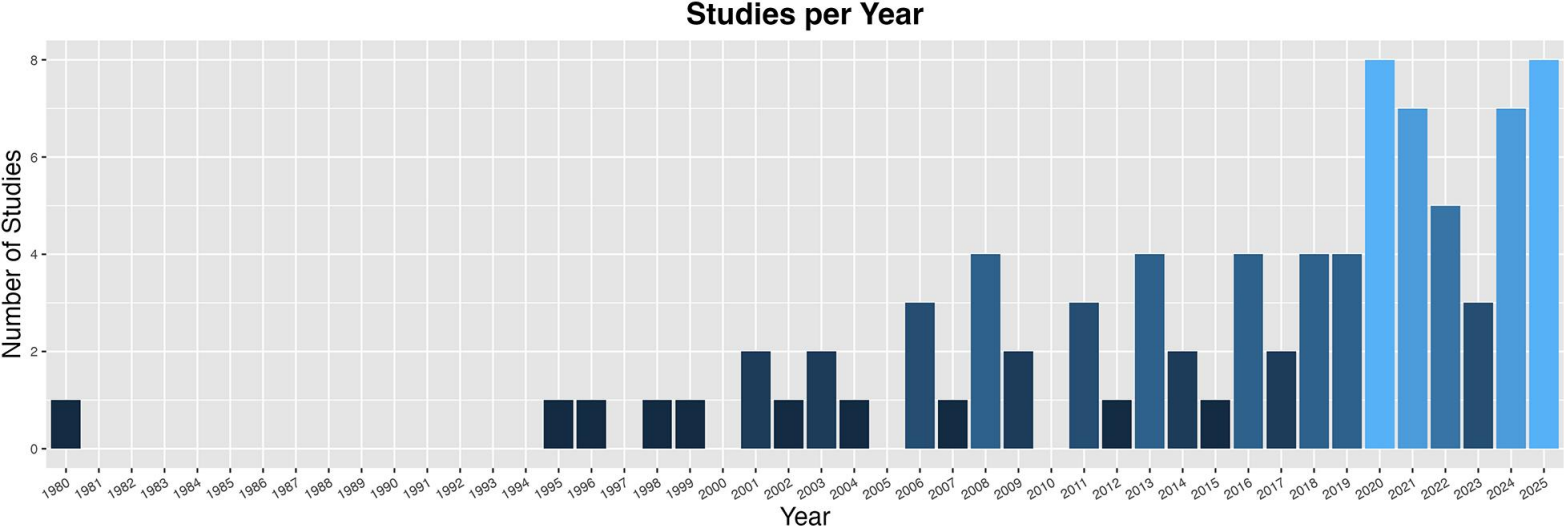
Number of Menopause symptom awareness studies published by year in the final sample.

The number of publications was then examined as a function of year. As can be seen from Figure 5, rates of publication have risen gradually over the years, a similar effect of overall global publication trends (28). Rate of publication particularly increased in the 2010's, potentially reflecting growing cultural awareness; for example, World Menopause Day was founded in 2009 (29). The Women's Health Initiative (WHI) 2002 study on menopause-related hormone replacement therapy (30) was also widely discussed and distributed by the media at the time and for years later (31), raising global interest in and awareness of menopause.

#### What levels of symptom awareness are reported by region and gender?

**Figure 6:**
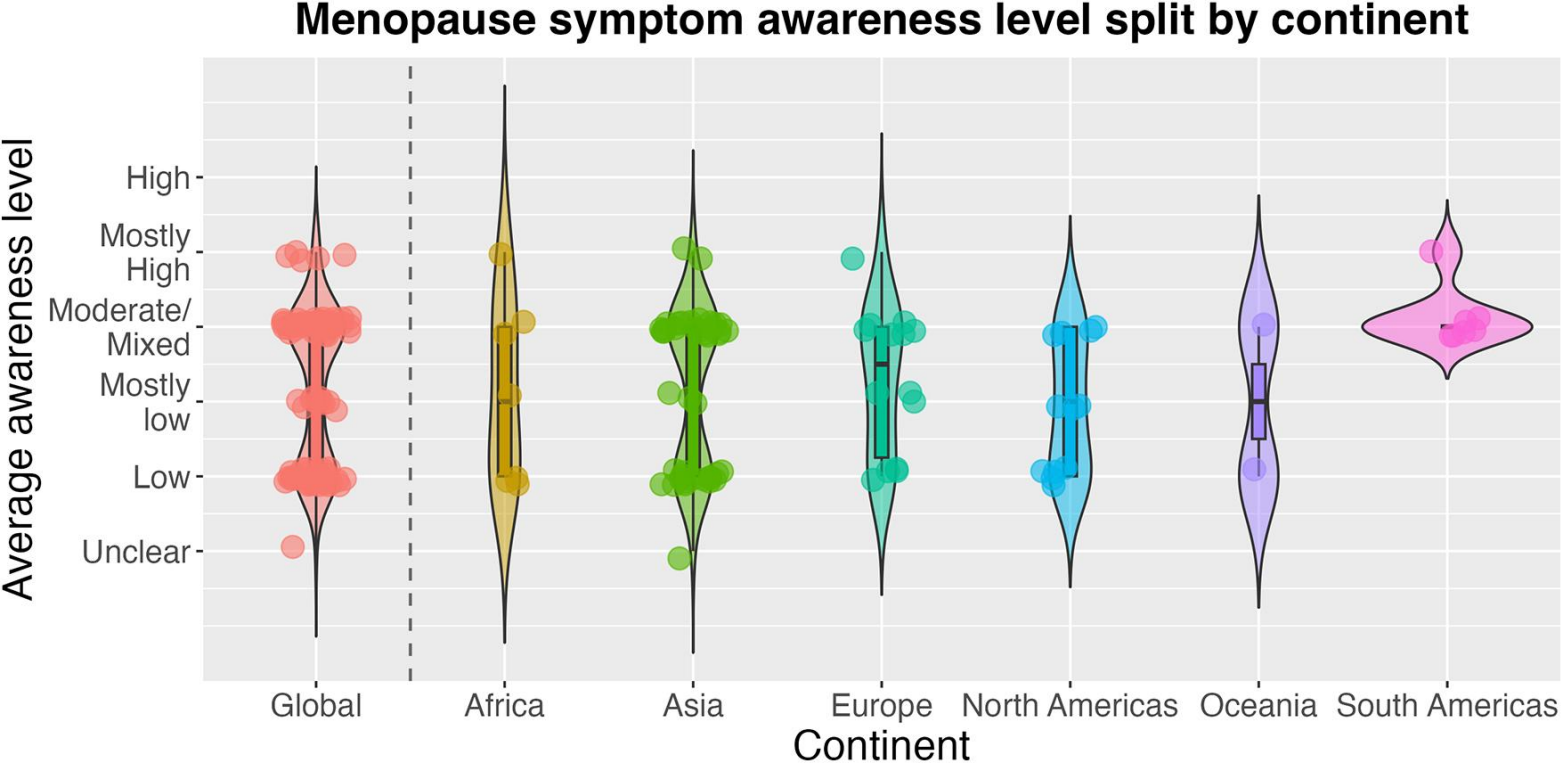
Coded average awareness level for each menopause symptom awareness study included in the final sample, split by continent. Global includes all studies, as a comparison. Jitter has been added to the datapoints to aid in visualisation.

Coded menopause symptoms awareness levels reported by each study, split by continent, are presented in Figure 6. After excluding studies with unclear average awareness levels, North America was found to demonstrate the lowest average symptom awareness levels (M[SD] = 1.9[0.9], *N* = 12), followed by Oceania (M[SD] = 2.0[1.4], *N* = 2), Africa (M[SD] = 2.1[1.2], *N* = 7), Asia (M[SD] = 2.2[1.0], *N* = 42), Europe (M[SD] = 2.3[1.0], *N* = 14), and South America (M[SD] = 3.1[0.4], *N* = 7), though the severely unequal number of studies limits comparison. The global average was 2.3 (SD = 1.0). It is notable that the results from none of the studies were graded as ‘high’ menopause symptom awareness.

**Figure 7:**
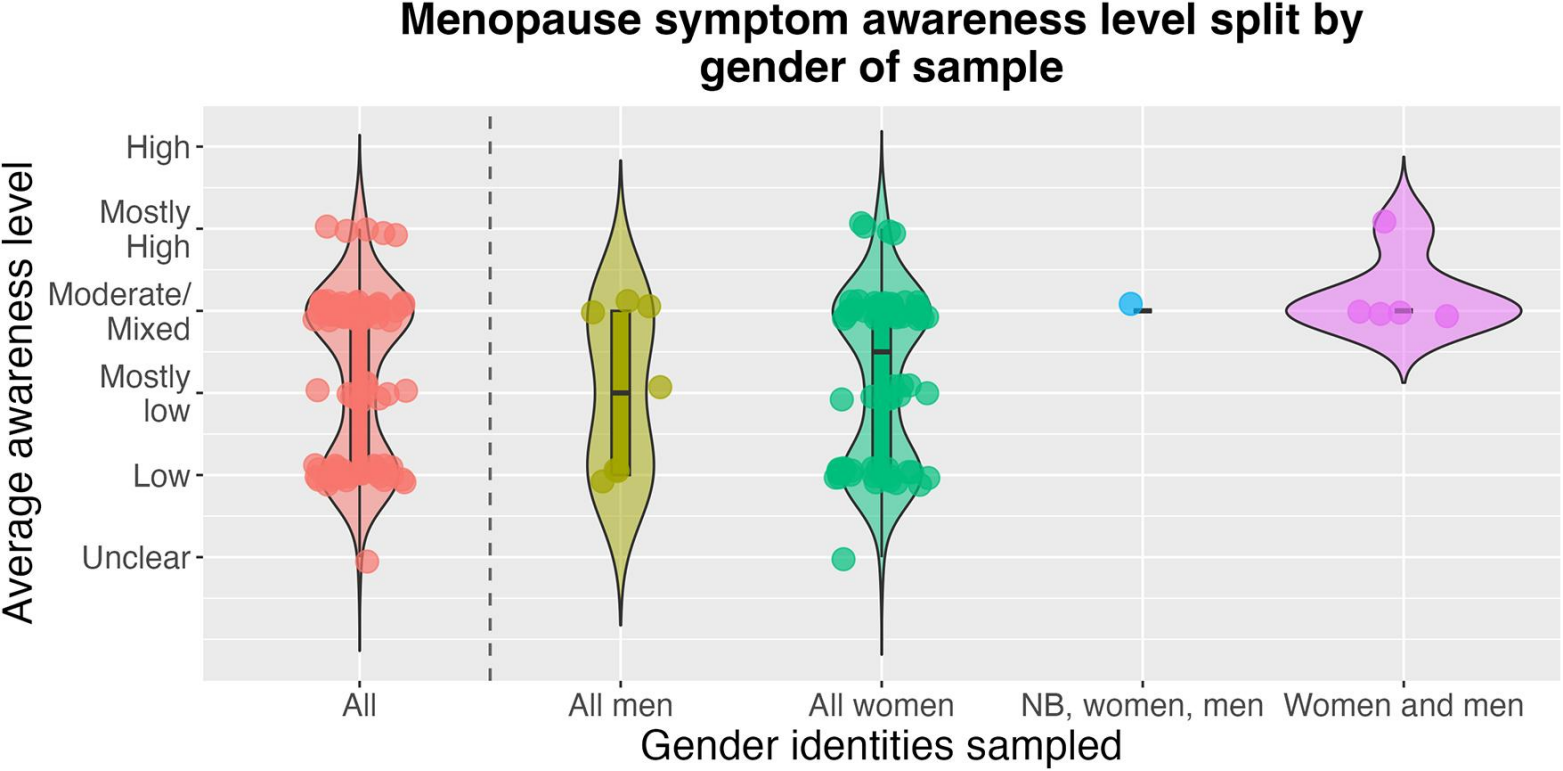
Coded average awareness level for each menopause symptom awareness study included in the final sample, split by gender composition of the study sample. ‘All’ includes all studies, as a comparison. NB, reported non-binary gender identity. Jitter has been added to the datapoints to aid in visualisation.

This was also examined across reported gender of the samples (results presented in Figure 7). After excluding ‘unclear’, we found that samples with both men and women had the highest reported awareness (M[SD] = 3.2[0.4], *N* = 5), followed by samples with all women (M[SD] = 2.2[1.0], *N* = 71), and finally samples with all men (M[SD] = 2.0[1.0], *N* = 7).

## Discussion

This scoping review aimed to map out the scientific landscape investigating menopause symptom awareness across cultures, genders, ages, and menopause status. We identified 84 primary studies published between 1980 and 2025. Our findings indicate major global gaps in menopause awareness research, which limits generalisability of knowledge and ability to target and tailor public awareness campaigns.

Our main research aim was to map what regions worldwide are represented in the current research base. A lack of research in the global South was identified, which mirrors other scientific domains [e.g., (25–27),]. The majority of research conducted about menopausal symptom awareness occurred in Asia, primarily Iran, India and Pakistan, and North America, particularly within the United States of America. African and Oceania countries were particularly poorly represented, which was reflected in the income analysis; there was a strong bias for studies to be conducted in higher-income countries compared to lower, potentially due to health priorities, low funding, and lack of access to menopause education and training in low- and middle-income countries (32).

Mapping symptom awareness across different African countries is vital to provide tailored and maximally beneficial awareness campaigns, especially considering the cultural, genetic, and symptomatology diversity across the continent (33–36). Women in African countries enter menopause at an earlier age compared to global averages (37) and frequency of certain symptoms differ across the world (38). Out of the African studies identified by this review, only one, which specifically sampled women working in education, reported mostly high awareness (39). It is also notable that Oceania was severely underrepresented, with no studies sampling Pacific Island communities. This reflects the wider lack of research into menopausal women's experiences from this region. Clinicians have reported poor knowledge, reluctance to discuss menopause, and poor accessibility as barriers to menopause treatment in the Asia-Pacific region (40). Improving understanding and ability to recognise menopausal symptoms is imperative for women to seek help, feel supported, and to avoid negative self-image and social exclusion in both the workplace and personal life (41–45).

We also examined the spread of sample characteristics across the included studies. The vast majority was found to focus on women-only samples, with most of these including menopausal women. This is obviously a critical target population - high awareness of potential menopause symptoms is vital for recognising and seeking help for these symptoms and may avoid anxiety if they can attribute symptoms to the menopause transition rather than other potential causes (e.g., dementia). However, for women to feel better prepared for the menopausal transition in terms of knowledge and support, both socially and in the workplace, it is important to extend knowledge and awareness education to other age groups and genders. Samples with men, non-binary individuals, and young women are under-represented in the scientific literature and should be included in the narrative going forward, particularly in these areas where cultural taboo may be shaping poor health literacy. Additionally, this is important to consider cross-culturally, as the perceived support and typical source of support (e.g., female family members, husbands, blood relatives, friends) differs across countries (42).

Another area of stark under-representation was the knowledge of transgender individuals, which was not explicitly included in any study [and gender diversity only reported in one (15);. Transgender individuals may present unique challenges and symptomatology across menopause due to previous use of hormone replacement therapies and potential unsettling of gender identities (46). It is important that any targeted awareness campaigns should be developed with members of the community (46, 47). In an exploration of transgender and gender non-conforming menopause experiences, people report a lack of inclusive representation surrounding menopause education, had a lack of knowledge themselves, and emphasised the need for more inclusive care (48).

Regarding menopausal symptoms, these findings reveal that there was a severe lack of research addressing ocular symptoms awareness, with only four studies (4.8% of the final sample) explicitly testing for them. Of those that did examine these symptoms, low awareness was reported compared to other domains (e.g., hot flashes, irregular menstruation) (15, 49–51). Despite this, such ocular symptoms are common in the menopausal population, with a reported prevalence of ∼60%–80% of dry eye in perimenopause (18, 19), which can be easily treated with eye drops if healthcare access is available [e.g. (52),]. However, they are often missed in global symptom mapping efforts. Investigating prevalence, awareness, and presentation of ocular symptoms should be a key area for future research. Finally, it should be noted that while other symptom domains were more likely to be explicitly mentioned in our sample (e.g., physical, vasomotor, psychological; for more examples, see Table 1), this still represents only a relatively limited breadth of research and only 56% of selected papers reported specific symptom domains. Thoroughly mapping awareness across the menopause experience across countries, especially when prevalence of such symptoms may vary per country (38), is vital to tailor awareness campaigns and understand regional needs.

Finally, reported symptom awareness levels were numerically coded and compared across studies. A range of levels were found, though it was notable that none were graded as ‘high’ menopause symptom awareness, highlighting the global struggle in this area. However, this comparison is limited due to the range of scales and methodologies used, exact details of which were not always readily available. This was particularly evident for qualitative studies, which offer a wealth of nuanced, in-depth information, but they were more likely to be marked unclear due to the difficulties in translating this to a numerical score and clarifying what symptom domains were included. While a small number of cross-cultural studies were identified (53–55), a more diverse and representative sample, with standardised testing, would allow for more informative cross-cultural and geographical comparisons to be made.

One limitation to our study design was the exclusion of articles not available in English language. Whilst automated translation tools have become more commonplace (for instance browser-based translators), it was decided not to use them for this review, due to the risk of losing nuance, which was particularly pertinent owing to the culturally sensitive nature of this topic. Since geographic coverage of menopause research was at the heart of this work, we wanted to ensure these pieces of research were not lost entirely. We therefore looked at a breakdown of studies excluded for this reason in the full-text screening stage (*n* = 22) by continent, and found the majority to be European (*n* = 11) followed by South American (*n* = 7), North American (*n* = 3), and Asian (*n* = 1). This did not affect our conclusion that Africa and Oceania remain particularly underrepresented in menopause awareness research. Additionally, while the screened databases were chosen for their breadth of coverage, it is possible that some relevant studies were missed.

## Conclusion

This scoping review highlights the urgent need for menopause symptom awareness research to expand beyond the global North, include more diverse populations, and assess thorough symptomatology. Menopause is not only a biological and medical transition– it also occurs within a social and cultural context whereby stigma and taboo regarding menopause can limit awareness and support while perpetuating health inequalities. This scoping review also draws attention to the need for more menopause education among general populations globally to combat stigma and generate more compassion towards menopausal individuals.

## Data Availability

The original contributions presented in the study are included in the article/Supplementary Material, further inquiries can be directed to the corresponding author.
