# Supplementary material for: Menopause symptom awareness research across the globe: a scoping review
Source: Front Glob Womens Health. 2026 May 28;7:1829378. doi: 10.3389/fgwh.2026.1829378 (PMC13253697; doi:10.3389/fgwh.2026.1829378)
Supplement: Supplementary file 1 [file Table1.docx]

**Menopause symptom awareness research across the globe: a scoping review.**

**Supplemental material**

*Data extraction sheet headings*

The following headings were used for data extraction purposes, with a separate row per study. The ‘Country code’ and ‘Continent’ columns were repeats as many times as required for that study (i.e., the value in the ‘Number of countries included’ column).

Table S1 – Data extraction sheet headings. Dropdown menu options are given for the headings if they were used to help with standardisation across the studies.

| **Heading** | **Dropdown menu options** |
| --- | --- |
| *Study ID* |  |
| *First author* |  |
| *Year* |  |
| *DOI* |  |
| *Country code* |  |
| *Continent* |  |
| *Number of countries included* |  |
| *Sample size* |  |
| *Majority age group* | Adolescents (<18), Young adulthood (18-25), Mid-reproductive life (25-40), Late reproductive life (40-60), Older adulthood (>60), Lifespan (evenly samples at least three) |
| *Age range* |  |
| *Gender identities included* | All women, All men, Women and men, Non-binary, women and men, Not reported |
| *Transgender identity* | All cisgender, All transgender, Mix, Not reported |
| *Socioeconomic/education status* | All high, Mostly high, All moderate, Mostly moderate, Equal mix, Mostly low, All low, Not reported |
| *Menopause status* | All peri/post-menopausal, Menopausal included, None menopausal, Status not defined |
| *Menopause definition* |  |
| *Specific symptoms assessed?* | Yes, No, Unclear |
| *Vasomotor* | Yes, No, Unclear |
| *Psychosocial* | Yes, No, Unclear |
| *Physical* | Yes, No, Unclear |
| *Sexual* | Yes, No, Unclear |
| *Ocular* | Yes, No, Unclear |
| *Quantitative/Qualitative* | Qualitative, Quantitative, Mixed |
| *Methodology* |  |
| *Scale used* |  |
| *Awareness level* | High, Mostly high, Moderate, Mostly moderate, Low, Mostly low, Mix, Unclear |
